# 529 Clinical Performance of Synthetic Dermal Skin Substitute in 77 Patients

**DOI:** 10.1093/jbcr/iraf019.158

**Published:** 2025-04-01

**Authors:** Sigrid Blome-Eberwein, Laetitia Roegner, Kurt Glaeser, Hamed Amani, Kyle Shaak

**Affiliations:** Lehigh Valley Health Network; Washington and Lee University; Lehigh Valley Health Network; Lehigh Valley Health Network; Lehigh Valley Health Network

## Abstract

**Introduction:**

Dermal skin substitutes are intended to compensate for the absence of dermal elements in traumatic wounds including severe burn injuries and are increasingly utilized in American Burn Centers. However, publications on the clinical performance of synthetic skin substitutes remain limited to small series or case reports. Furthermore, scarring outcomes after the two-step process of applying synthetic skin substitutes and split-thickness skin grafts (STSG) have hardly been studied at all. This IRB approved study was designed as a retrospective detailed assessment of the performance of synthetic skin substitutes, scarring outcomes, and their overall effectiveness in our center.

**Methods:**

This study included all patients who underwent the two-stage wound closure procedure between 9/1/2017 and 2/28/2024. Patient electronic records were reviewed retrospectively. The evaluated data points included complications, as determined by infection and failure rates, the time delay between the application of the synthetic skin substitute and STSG, LOS after skin substitute placement, Vancouver Scar Scale (VSS) assessments, Patient and Observer Scar Assessment Scale (POSAS) questionnaires, and scar shrinkage measurements during follow up visits. All data were entered into RedCap® for analysis.

**Results:**

The study included 77 patients and a total of 124 dermal synthetic skin substitute applications (122 synthetic biodegradable, 2 collagen based). All applications were analyzed, and the infection rate overall was 8.8%. The failure rate was found to be 3.2%. The time delay between the application date of the skin substitute and STSG averaged 28 days, to wound closure 35 days. The LOS after skin substitute averaged 45 days, after wound closure 10 days. Data on postoperative dressings (Silver 54.8%, VAC 38.7%), STSG thickness (0.0085), exposed structures in the wound etc. were also collected. 3-12 months post hospital discharge, the mean Vancouver scar scale rating was 8.39 out of 15, the mean Patient and Observer Scar Assessment Scale rating was 36.86 out of 60, and the median scar shrinkage was 2.89% of the initial wound size.

**Conclusions:**

The usage of synthetic dermal skin substitute allows for a safe, reliable two-stage burn and wound closure. This burn/wound treatment exhibited minimal complications and long-term durability with acceptable scar formation and shrinkage.

**Applicability of Research to Practice:**

Immediate

**Funding for the Study:**

Institutional Burn research fund

